# Purple Urine Bag Syndrome: A Peculiar Presentation of a Urinary Tract Infection

**DOI:** 10.7759/cureus.49804

**Published:** 2023-12-01

**Authors:** João Faia, Ana S Martins, Miguel Martins

**Affiliations:** 1 Internal Medicine, Centro Hospitalar do Baixo Vouga E.P.E, Aveiro, PRT

**Keywords:** urinary catheter, pubs, uti, urinary tract infection, purple urine bag

## Abstract

Purple urine bag syndrome (PUBS) is a peculiar phenomenon and corresponds to the appearance of purplish-colored urine. It is associated with urinary tract infections occurring mainly in debilitated elderly women with constipation and long-term indwelling urinary catheters. We share a case involving PUBS in an 87-year-old female patient, explore the pathophysiology, and discuss potential management options for this uncommon syndrome.

## Introduction

Purple urine bag syndrome (PUBS), a rare phenomenon initially documented in 1978, derives its name from the distinctive occurrence of purple-colored urine [1]. It is a condition often associated with a urinary tract infection (UTI), especially in females, dependent and with long-term indwelling urinary catheters [1,2].

This article was previously presented as a poster at the 20th European Congress of Internal Medicine on June 9-11, 2022.

## Case presentation

The patient was an 87-year-old female who was institutionalized, dependent for activities of daily living, and bedridden most of the day. Her personal history included Parkinson's disease, stroke, dyslipidemia, hypertension, hypothyroidism, and hearing loss. In addition, in the past, there were multiple episodes of urinary retention requiring a chronic indwelling Foley catheter. She was medicated with acetylsalicylic acid 100 mg, ramipril 5mg, selegiline 5mg, simvastatin 20 mg, and levothyroxine 0.05 mg. She was admitted to the emergency department due to a two-day history of changing mental status.

**Figure 1:**
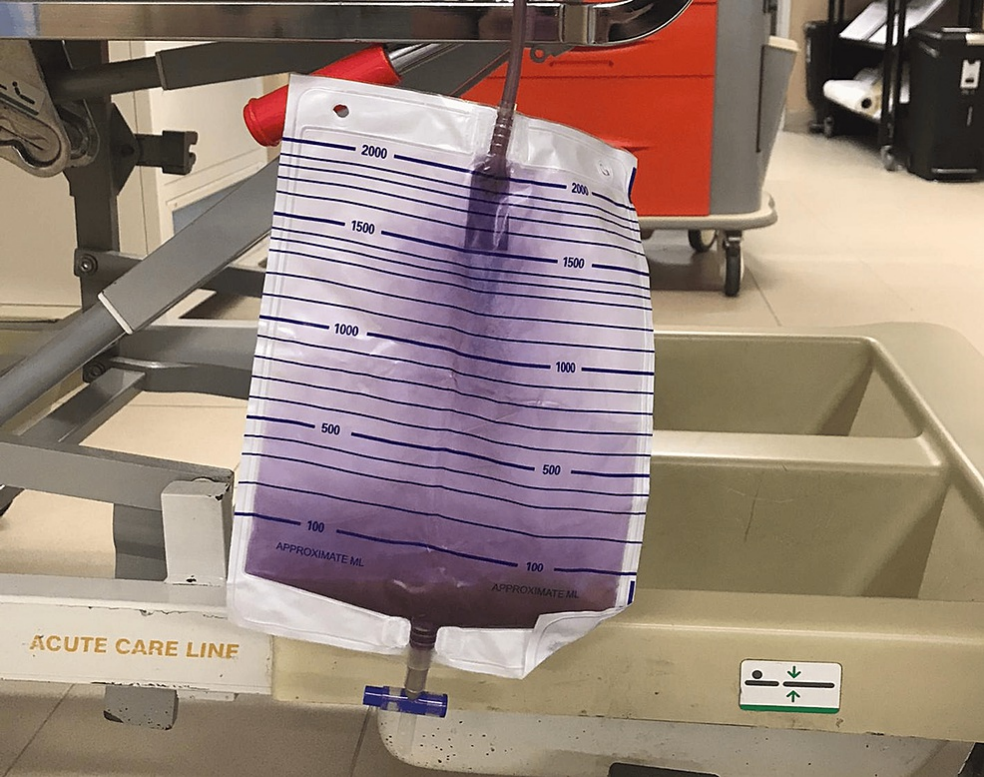
Collector Bag with Purple Urine

The physical examination uncovered the existence of urine with a purplish hue in the collection bag (Figure 1), without other noteworthy findings.

Blood tests were carried out which showed a slight increase in C-reactive protein (Table 1) and urinalysis was positive for nitrites and leucocyte esterase, with high levels of leucocytes (100 white blood cells per high-power field) and alkaline urine (Table 2).

**Table 1:** Blood Test Results

Test	Value	Normal Range
Hemoglobin	12.3 X10E9/L	12.0 - 18.0 X10E9/L
Leucocytes	10.9 X10E9/L	4.1 – 11.1 X10E9/L
Platelets	200 X10E9/L	150 – 500 X10E9/L
Creatinine	0.89 mg/dL	0.6 - 1.1 mg/dL
Sodium	142 mEq/L	135 – 145 mEq/L
Potassium	4.2 mEq/L	3.5 – 5.2 mEq/L
Alanine transaminase	19 U/L	7 - 55 U/L
Aspartate transaminase	23 U/L	8 - 48 U/L
Lactate dehydrogenase	150 U/L	122-222 U/L
Alkaline phosphatase	98 U/L	45-115 U/L
Gamma-glutamyltransferase	18 U/L	8- 61 U/L
C-reactive protein	7.8	0.3 – 1.0 mg/dL

**Table 2:** Urinalysis Results

Test	Value	Normal Range
Macroscopic Examination		
Color	Yellow	-
Turbidity	Turbid	-
Urine Chemical		
pH	7.7	5.00 - 7.00
Specific Gravity	1.013	1.005 - 1.025
Protein	Negative	Negative
Glucose	Negative	Negative
Bile	Negative	Negative
Urobilinogen	Negative	Negative
Blood	Negative	Negative
Leukocyte Esterase	Positive	Positive
Nitrite	Positive	Positive
Microscopic		
White Blood Cells	100 /hpf	0-5 /hpf
Red Blood Cells	Negative	0-3 /hpf
Epithelial Cells	Few	-

In view of the findings, hypoactive delirium in the context of a UTI was assumed. The Foley catheter was changed and empirical amoxicillin with clavulanic acid at a dosage of 1,2 g was started. The urine culture indicated the presence of Providencia stuartii and given the results of the antibiogram, the antibiotic was changed to oral cefixime 400 mg which was continued for seven days with favorable evolution.

## Discussion

PUBS represents an uncommon presentation of a UTI, with reported prevalence reaching as high as 9.8% in institutionalized individuals utilizing long-term indwelling urinary catheters [2-4].

This phenomenon results from the metabolism of tryptophan by the intestinal microbiota, originating indole, which in turn is conjugated into indoxyl sulfate in the liver. The majority of indoxyl sulfate is eliminated in the urine. Bacterial enzymes, specifically sulfatases and phosphatases, play a role in the conversion of indoxyl sulfate into indirubin and indigo. Indirubin is red, and indigo is blue; when these pigments are combined, they create a purplish color [1,5].

The prevalent microbes implicated in PUBS-associated UTIs include Escherichia coli, Enterococcus, Proteus mirabilis, Klebsiella pneumoniae, Providencia stuartii, Pseudomonas aeruginosa, Morganella morgannii, and others [5].

A plethora of factors are associated with this phenomenon. The most frequently described were female gender, advanced age, bedridden situation, dementia, constipation, institutionalization, dehydration, end-stage renal disease, chronic catheterization, use of a polyvinyl chloride urinary catheter or bag, alkaline urine, recurrent UTIs, and high urinary bacterial counts [1,5]. Among these factors, chronic constipation is perhaps one of the most important, being described in around 90.1% of cases according to some systematic reviews [5]. The hypothesis is that diminished gut motility and prolonged transit time contribute to bacterial overgrowth in the bowel lumen, facilitating the conversion of tryptophan to indole [5,6]. Besides that, the use of laxatives may lead to damage of the colorectal mucosa and changes of normal intestinal microbiota [7].

Despite being ostentatious, PUBS is a relatively harmless occurrence. Nevertheless, it might not consistently manifest as a benign condition and could be linked to important morbidity and mortality [8,9]. In fact, there are some cases of PUBS with progression to Fournier's gangrene and necessity of aggressive debridement [10].

Treatment involves starting antibiotic therapy, in cases where infection is assumed, and replacing the Foley catheter with a new bag. Furthermore, it is necessary to intervene in the various predisposing factors, namely constipation, good urological hygiene, avoiding chronic indwelling catheters, and employing periodic catheter exchanges [1,11,12].

## Conclusions

PUBS is a peculiar situation associated with UTIs, causing anxiety to families, caregivers, and healthcare workers who are unaware of this phenomenon. It mainly affects frail, bedridden, chronically catheterized, and constipated elderly patients.

With this clinical case, we intend to raise awareness of this phenomenon, especially among doctors who come into contact with the geriatric population, and provide a correct approach to this condition involving not only the appropriate use of antibiotics but also preventive measures, namely, the treatment of constipation and rational use of urinary catheters.
